# Evaluation of MT Family Isoforms as Potential Biomarker for Predicting Progression and Prognosis in Gastric Cancer

**DOI:** 10.1155/2019/2957821

**Published:** 2019-07-17

**Authors:** Mingfu Tong, Wenquan Lu, Hao Liu, Jian Wu, Mingzuo Jiang, Xin Wang, Jianyu Hao, Daiming Fan

**Affiliations:** ^1^Department of Gastroenterology, Beijing Chao-Yang Hospital, Capital Medical University, Beijing 100020, China; ^2^Department of Gastroenterology, First Affiliated Hospital, Zhengzhou University, Henan, Zhengzhou 450000, China; ^3^State Key Laboratory of Cancer Biology and Xijing Hospital of Digestive Diseases, Xijing Hospital, Fourth Military Medical University, Xi'an 710032, China

## Abstract

**Background:**

Metallothioneins (MTs) family comprises many isoforms, most of which are frequently dysregulated in a wide range of cancers. However, the expression pattern and exact role of each distinct MT family isoform which contributes to tumorigenesis, progression, and drug resistance of gastric cancer (GC) are still unclear.

**Methods:**

Publicly available databases including Oncomine, Gene Expression Profiling Interactive Analysis (GEPIA), Kaplan-Meier plotter, SurvExpress, MethHC, cBioportal, and GeneMANIA were accessed to perform an integrated bioinformatic analysis and try to detect fundamental relationships between each MT family member and GC.

**Results:**

Bioinformatic data indicated that the mRNA expression of all MT family members was almost lowly expressed in GC compared with normal gastric tissue (P<0.05), and patients with reduced mRNA expression of each individual MT member had inconsistent prognostic value (OS, FP, PPS), which depended on the individual isoform of MT. A negative correlation between the methylation in promoter region of majority of MT members and their mRNA expression was detected from MethHC database (p<0.001). Data downloaded from TCGA revealed that MTs were rarely mutated in GC patients and MT2A was frequently regulated by other three genes (FOS, JUN, SP1) in GC patients.

**Conclusion:**

MTs were nearly downregulated, and distinct type of MT harbored different prognostic role in GC patients. Methylation in gene promoter region of MTs partially contributed to their reduced expression in GC. Our comprehensive analyses from multiple independent databases may further lead researches to explore MT-targeting reagents or potential diagnostic and prognostic markers for GC patients.

## 1. Introduction

Epidemiological data from the WHO suggested that gastric cancer (GC) is the fifth most common malignant tumor and the third leading cause of cancer related death throughout the world, with 1,033,701 new cases and 782,685 deaths in 2018 [1]. Despite a decline rate in incidence and important advances in understanding of the epidemiology, pathology, molecular mechanisms, and treatment options made, the disease was still among the poorest of all solid-organ tumors, predominately due to the frequent presence of advanced stage of the cancer once at first diagnosis [2]. In order to improve the survival of advanced GC patients, based on palliative surgery and chemotherapy, targeted therapy had been introduced and was expected to be an important supplementary treatment for gastric cancer [3]. Furthermore, exploring new highly specific and sensitive biomarkers and new molecular targets can not only improve the prognosis of GC patients, but also help to elucidate the molecular mechanism of GC.

Metallothioneins (MTs) are a group of high conserved, low molecular metal-binding proteins with a high content of cysteinyl residues that had been found in bacteria, plants, invertebrates, and vertebrates [4]. In mammals, MTs are clustered on chromosome 16 and encode four protein isoforms whose amino acids varying from 61 to 68, labelled by numbers: MT1, MT2, MT3, and MT4 [5]. Despite the physical and chemical similarity of MT isoforms, their roles and presence in tissues vary significantly. MT1 comprises eight functional paralogs, named MT1A, MT1B, MT1E, MT1F, MT1G, MT1H, MT1M, and MTX, present almost in all types of soft tissue [6]. MT2 gene only encodes one isoform, called MT2A, also existing prevalently like MT1. MT3 and MT4 are both encoded by one single gene, whereas they are expressed respectively in brain tissues and epithelial cells [6]. Although abundant researches appeared, the proper functions of MTs are still illusive. Nevertheless, MTs had been implicated in a wide range of properties like homeostasis maintenance, detoxification, DNA damage protection, redox pool maintenance, inflammation, and cancer regulation [7].

It is not surprising that MTs are involved in many cancer processes, but the expression and role of MTs is not uniform in kinds of malignancy [8–11]. Discordant results regarding the expression of MT and its association with clinicopathological parameters and prognosis were observed in gastric cancer tissue compared to normal tissue in different studies [12–20]. In addition, the change of MT1/2 protein expression differs from the change of single MT isoform in malignant melanoma tumor, like MT1E and MT1G [21–23]. As such, it is urgent to systematically investigate the expression and role of each isoform of MTs in gastric cancer. In the present study, we accessed into some available databases, like Oncomine, Gene Expression Profiling Interactive Analysis (GEPIA), Kaplan-Meier plotter, SurvExpress, MethHC, cBioportal, and GeneMANIA to systematically evaluate MT family isoforms in gastric cancer, which may be able to pave the way to well-understand the expression and role of MTs in gastric cancer.

## 2. Materials and Methods

All datasets obtained from various public databases were analyzed to predict MTs mRNA expression levels, prognostic values, methylation and mutation of metallothionein in tumor tissue compared to normal gastric mucosae.

### 2.1. Comparison of MTs Gene Expression between Tumor and Normal Samples

The cancer related public databases Oncomine (https://www.oncomine.org/) was used to investigate the mRNA expression level of MTs in tumor and normal tissue [24]. In the Oncomine database, all members of MT family were retrieved and the differential gene analysis (GC versus normal) combined with mRNA data type were chosen. In this study, the Student's t-test was used to generate* p* values of comparison. The cutoff* p* value and fold change were defined as 0.01 and 2.

The expression of MTs between tumor and normal gastric tissue was also studied using the GEPIA browser (http://gepia.cancer-pku.cn/), which is an online tool for estimating mRNA expression based on The Cancer Genome Atlas (TCGA) and the Genotype-Tissue Expression (GTEx) projects [25]. Box and stage plotting analyses were processed on this database. The cutoff* p* value was defined as 0.01.

### 2.2. Analysis of Prognostic Values of MT Members in GC Patients

The association among MTs expression and the overall survival (OS), first progression (FP), and postprogression survival (PPS) in GC was analyzed by data mining in the Kaplan-Meier plotter database (http://kmplot.com), which is an online database that enables assessment of survival related biomarkers download from Gene Expression Omnibus (GEO) [26]. In this study, clinical data including subtypes, stage, differentiation, HER2 status, and treatment was collected.

SurvExpress (http://bioinformatica.mty.itesm.mx:8080/Biomatec/SurvivaX.jsp), a large online database that enables comparison and validation of survival related biomarkers for cancer outcomes was used when the survival data of some MT family members were not available in Kaplan-Meier plotter [27]. The parameters chosen for survival analysis were as follows: larger stomach adenocarcinoma (STAD) sample size (>200 patient), dataset from TCGA, duplicated genes-show all, data-uniformized. The median MTs expression was used as the cutoff. Hazard ratios with 95% CI and log-rank p value were calculated.

### 2.3. Comparison of MTs Gene Methylation between Tumor versus Nontumor Tissues and Analysis of Relationship between Methylation and mRNA Expression in GC from MethHC

DNA methylation of MTs between tumor and normal tissue was compared through the human pan-cancer methylation database-MethHC (http://methhc.mbc.nctu.edu.tw/), which is a database focused on the DNA methylation of human diseases from TCGA [28]. In addition, the correlation between MTs methylation and its mRNA expression in GC patients was also analyzed using MethHC. In this study, the gene region was chosen as promoter and the methylation level evaluation method was defined as average.

### 2.4. Analysis of MTs Gene Mutations and Associated Network in GC from TCGA

Clinical data from TCGA database for GC patients were downloaded and processed in Microsoft Excel and manually checked on the base of the primary site of tumor onset in a bid to exclude non-GC patients. Meanwhile, the information of GC downloaded in the cBioPortal for Cancer Genomics (http://www.cbioportal.org) was processed to analyze the presence of mutations, EBV infection rate, and explore the associated network of MTs in GC [29, 30]. GeneMANIA, a flexible, accurate database that can generate network information based on genes inputted including protein and genetic interactions, pathways, coexpression, colocalization, and protein domain similarity [31], was used to find additional genes or proteins related to MTs.

## 3. Results

### 3.1. Downregulation of MTs mRNA in Patients with GC

**Figure 1:**
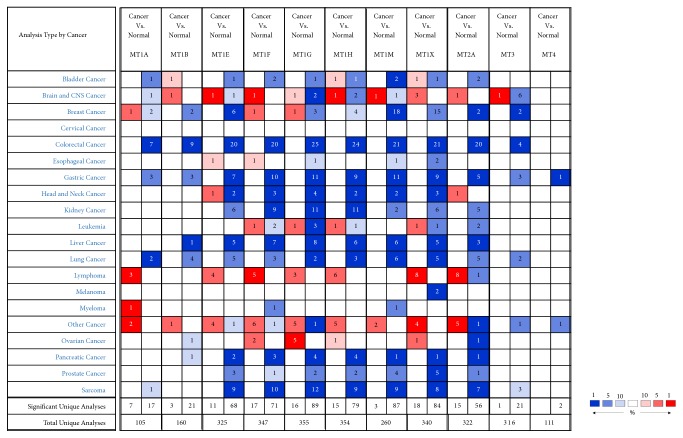
Transcript levels of MT isoforms in different types of cancer (Oncomine).* Notes:* This figure indicates the numbers of datasets with statistically significant MTs mRNA upregulation (red) or downregulation (blue) (different types of cancer versus corresponding normal tissues) (threshold setting: p value, 0.05; fold change, 2; gene rank, top 10%). The numbers in the colored cell represent the numbers of dataset meeting the threshold.

**Figure 2:**
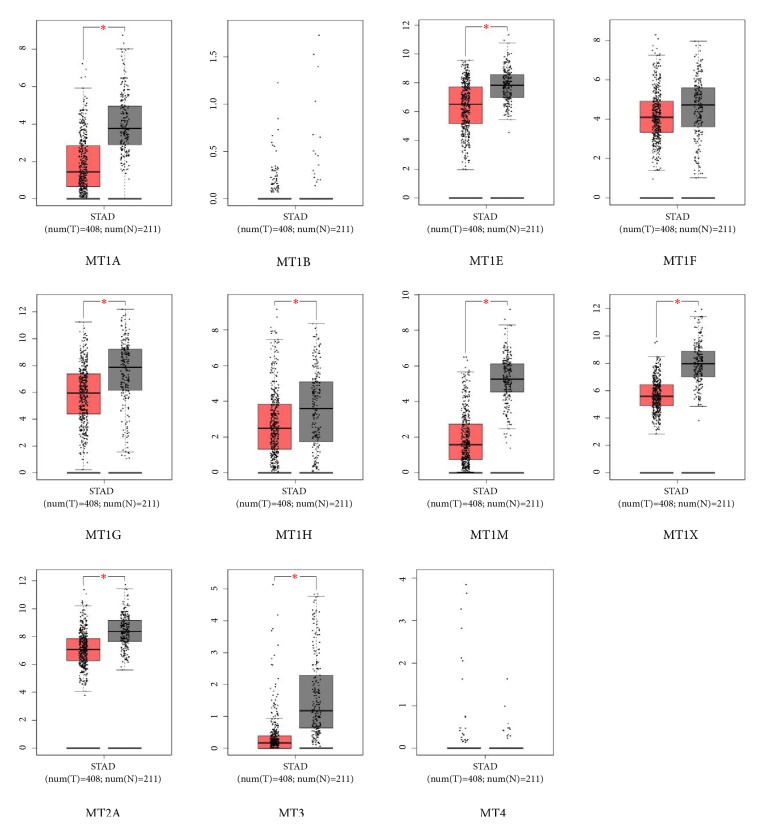
The distinct expression of MT family isoforms between cancer and normal tissues in GC patients (GEPIA).* Notes:* Box plots derived from gene expression data in GEPIA comparing expression of a specific MT isoform in GC tissue and normal tissues; the P value was set up at 0.05.* Abbreviations:* GC: gastric cancer; STAD: stomach Adenocarcinoma; T: tumor; N: normal.

Oncomine and GEPIA databases data was used to examine differential levels of MTs mRNA between gastric cancer and normal gastric tissue. In addition to GC, difference of MTs mRNA in other cancers and their paired normal tissue was also assessed in Oncomine database. Among these cancer datasets, the expression of all MT isoforms was downregulated significantly in 8 out of 20 cancer types compared to paired normal tissue, including four digestive system cancer types: gastric, colorectal, liver, and pancreatic cancer (Figure 1). Apart from MT1B, MT1F, and MT4, other MT isoforms in tumor tissue were both downregulated significantly in Oncomine and GEPIA databases (Figures 1 and 2). The elaborating details of MTs expression in all GC datasets in Oncomine database could be seen in Table 1. In addition, the expression of MT family members in different stages of GC was also analyzed using GEPIA, and none of them varied with statistical significance in different stages of GC (Supplementary Figure 1).

### 3.2. Prognostic Features of MTs in Patients with GC

**Figure 3:**
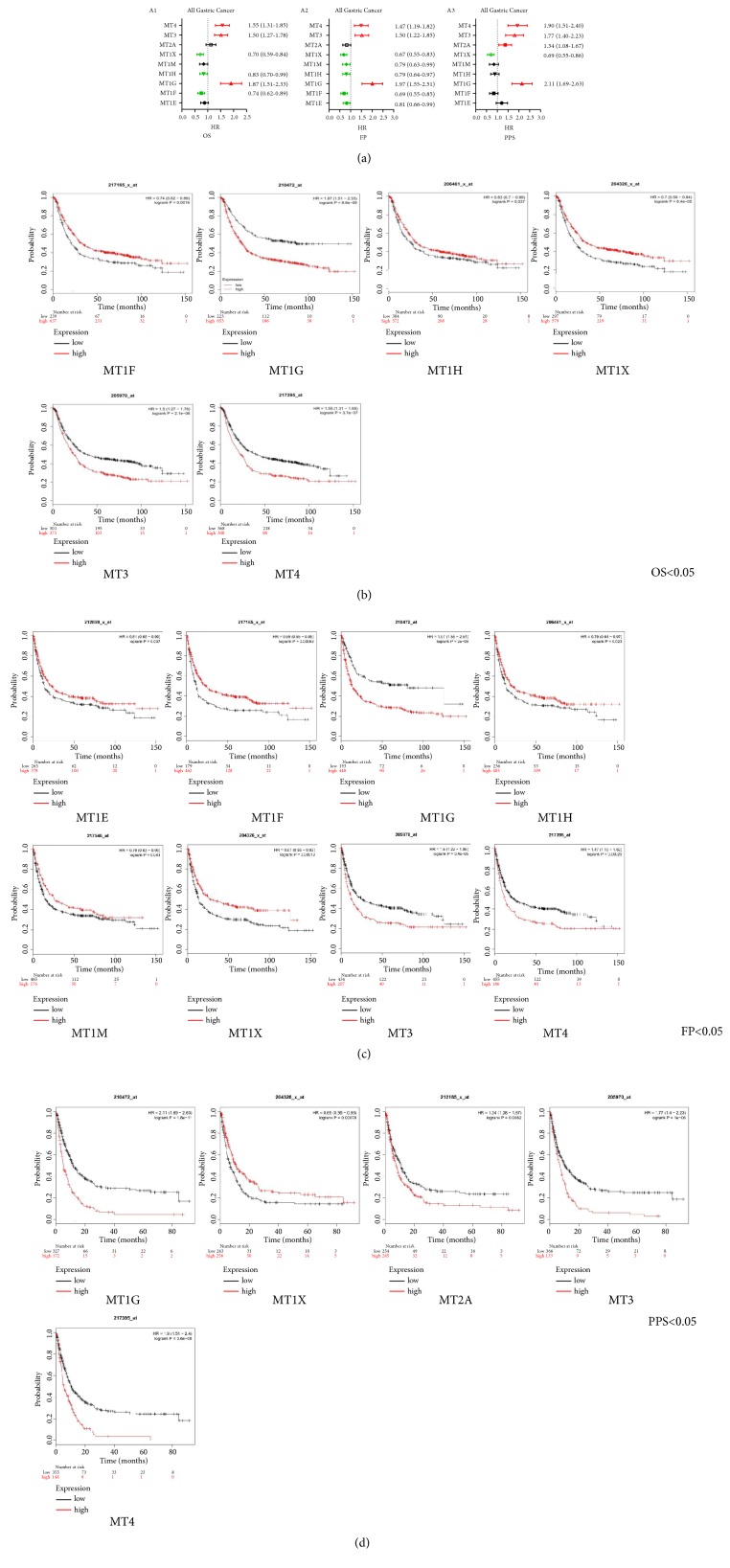
The prognostic values of mRNA level of MTs in all GC patients (Kaplan-Meier plotter).* Notes:* Kaplan-Meier plots show the association between the expression of MTs and OS, FP and PPS in GC patients, respectively, with statistical significance. A1–3: Prognostic HRs of individual MT isoform in all gastric cancer for OS, FP, and PPS. (b) OS curves of MT1F (Affymetrix ID:217165_-_x at); MT1G (Affymetrix ID:210472_-_x at); MT1H (Affymetrix ID:206461_-_x at); MT1X (Affymetrix ID:204326_-_x at); MT3 (Affymetrix ID:205970_-_x at); MT4 (Affymetrix ID:217395_-_x at). (c) FP Curves of MT1E (Affymetrix ID:212859_-_x at); MT1F; MT1G; MT1H; MT1M (Affymetrix ID:217546_-_x at); MT1X; MT3; MT4. (d) PPS curves of MT1G; MT1X; MT2A (Affymetrix ID:212185_-_x at); MT3; MT4.* Abbreviations:* OS: overall survival; FP: first progression; PPS: postprogression survival; GC: gastric cancer; HR: hazard ratio.

Prognostic features of MTs mRNA for GC patients including OS, FP, and PPS were investigated, respectively, through data mining in Kaplan-Meier plotter. It could be seen that almost all MTs prognostic feature can be searched out in GC patients other than MT1A and MT1B, both of which were analyzed alternatively by using SurvExpress database. There was no significant correlation in gastric cancer between OS and either MT1A or MT1B (supplementary Figure 2). Among these MTs available in the Kaplan-Meier, 6, 8, and 5 isoforms mRNA were significantly associated with OS, FP, and PPS for GC patients, respectively (Figure 3 A1–A3). The data from the respective probes showed reduced OS with low MT1F, MT1H, and MT1X (Figure 3(b)) and reduced FP with low MT1E, MT1F, MT1H, MT1M, and MT1X (Figure 3(c)). Positive correlation was found between PPS and MT1X, while reversed relationship was shown between PPS and MT1G, MT2A, MT3, and MT4 (Figure 3(d)). High MT1G, MT3, and MT4 mRNA expression led to reduced OS, FP, and PPS in GC patients. Notably, increased MT2A transcript level only correlated significantly with reduced PPS, not correlated significantly with OS and FP (Figures 3(b)–3(d)). The details of these isoforms whose mRNA expression was not correlated with survival time (OS, FP, PPS) were listed in supplementary materials (Supplementary Figures 2(A)–2(D)).

As per the Lauren's classification of stomach adenocarcinoma, GC was classified into three subtypes: intestinal type, diffuse type, and mixed type. As such, prognostic value of MTs isoforms was also determined in different GC subtypes using Kaplan-Meier plotter online tool. The data from individual probe indicated that 8 out of 9 available MT members mRNA expression were correlated with OS in GC intestinal type (P<0.05; Table 2). Furthermore, the majority of them (5/8) were with better prognosis (OS) (data was not shown). In addition, other survival analysis revealed that clinicopathological features including clinical stage, differentiation, HER2 status, and treatment were significant parameters affecting the survival time of GC patients (Table 3, supplementary Tables 1–3).

### 3.3. DNA Methylation of MTs and Its Correlation with mRNA Expression in GC Patients

**Figure 4:**
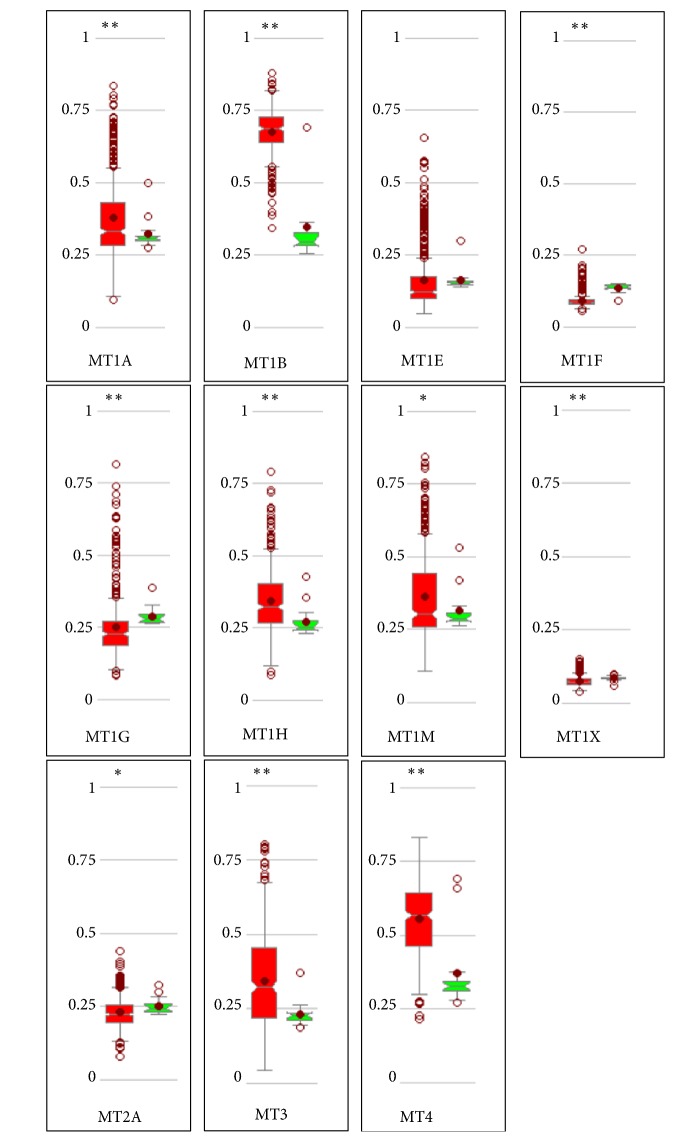
The distinct methylation of MT isoforms in promoter region between cancer and normal tissues in GC patients (MethHC).* Notes:* box plots in red color represent cancer samples and those in green color represent normal samples. GC: gastric cancer; “^*∗*^” indicates being statistically significant with* P*<0.05. “^*∗∗*^” indicates being statistically significant with* P*<0.005.

To identify the role of methylation in regulating MTs expression in patients with GC, MethHC was utilized to explore the level of methylation in promoter region and its relationship with mRNA expression of MT genes. Among all types of MT, the difference of methylation level between cancer and normal samples was statistically significant except gene MT1E (P<0.05, Figure 4). The majority of MTs (8/11) in cancer exhibited extraordinarily methylated variation in promoter region compared to normal tissue (P<0.005, Figure 4). Notably, DNA methylation of some MT isoforms in gastric cancer, like MT1A, MT1B, MT1H, MT1M, MT3, and MT4, was higher than their paired normal tissue except remaining isoforms (Figure 4). Additionally, an inverse correlation between DNA methylation and mRNA expression of most isoforms of MT in GC was observed other than MT1A and MT4 isoforms (P<0.001, Table 4).

### 3.4. MTs Mutations and Associated Network in GC Patients

**Figure 5:**
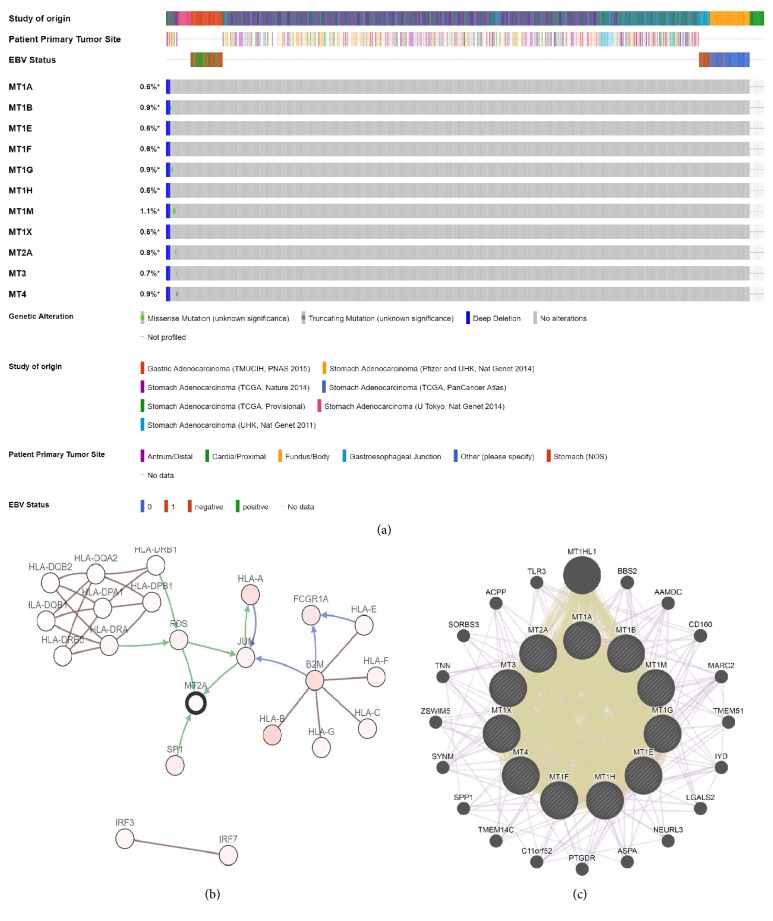
Alteration frequency of MT isoforms and neighbor genes network in GC patients (cBioPortal).* Notes:* (a) OncoPrint visual summary of alteration in MT family members. (b) Network involved in the expression of MTs gene constructed in cBioPortal. Green lines represent gene controlling the expression of those genes to which the arrows are pointing, while blue lines represent genes controlling the state change of those genes to which the arrows are pointing, brown lines represent genes in complex with other genes. (c) Network for MTs with the structure or neighboring genes constructed in GeneMANIA. Yellow lines represent shared protein domains between these genes; violet lines represent coexpression between these genes.

Genetic mutations of MT family members were analyzed through cBioPortal online tool for GC patients. A total of 1443 patients from seven datasets of stomach adenocarcinoma were analyzed. Among these seven datasets, mutation rate of MTs calculated in three datasets was 1.36% (6/440), 2.51% (12/478), and 3.39% (10/295), respectively, and no statistically significant difference was observed for OS and DFS between cases with and without MT mutation in gastric cancer (data was not showed). The percentage of genetic mutation in MT1A, MT1B, MT1E, MT1F, MT1G, MT1H, MT1M, MT1X, MT2A, MT3, and MT4 was 0.6% (deep deletion), 0.9% (0.21% missense mutation, 0.62% deep deletion), 0.6% (deep deletion), 0.6% (deep deletion), 0.9% (0.28% missense mutation, 0.62% deep deletion), 0.6% (deep deletion), 1.1% (0.42% missense mutation, 0.62% Deep Deletion), 0.6% (deep depletion), 0.8% (0.14% missense mutation, 0.62% deep depletion), 0.7% (0.07% truncating mutation, 0.62% deep depletion), and 0.9% (0.27% truncating mutation, 0.62 deep depletion) (Figure 5(a)). Data from in situ hybridization (ISH) revealed that 20% (40/200) were EBV positive. In addition, crossing data of primary site showed that 37.1% (161/434) of these tumors were located at the antrum, followed by fundus/body (35%), cardia/proximal (14.3%), gastroesophageal junction (10.6%), and the unknowable site (3%) (Figure 5(a)).

The network established in cBioPortal demonstrated that FOS, JUN, and SP1 control the expression of MT2A, whereas HLA-DRA and HLA-DRB1 control the expression of FOS. FOS controls the expression of JUN; meanwhile, B2M and HLA-B control the state change of JUN (Figure 5(b)). Furthermore, another network for MTs with the structure or function of neighboring genes constructed from GeneMANIA showed that other 20 genes—MT1HL1, BBS2, AAMDC, CD160, MARC2, TMEM51, IYD, LGALS2, NEURL3, ASPA, PTGDR, C11orf52, TMEM14C, SPP1, SYMM, ZSWIM5, TNN, SORBS3, ACPP, and TLR3—were associated closely with MTs. The result showed that all MTs protein shared protein domains with each other and particularly shared protein domains even with another protein named MT1H1 (Figure 5(c)).

## 4. Discussion 

Up to date, accumulating studies were emerging to investigate MTs expression and their roles in malignant tumors, but only a minority of MT isoforms were evaluated in gastric cancer and no unanimous agreement was reached. In an immunohistochemical analysis, Ebert and his colleagues showed overexpression of MT in GC tissues, independent of tumor stage, differentiation, or tumor type [14]. Similar outcome of MT in GC was also reported by other groups [17, 32]. On the contrary, several studies reported a lower MT expression in GC specimens than normal mucosae [15, 16, 18]. Until now, a minority of individual isoform of MTs involved in GC was reported, such as MT1A, MT2A, and MT3 [13, 14, 19, 20, 33, 34]. The upregulation of MT3 in GC of one individual study was consistent with the result demonstrated in our current study [33]. MT2A expression in GC reported by pan's group was in accordance with the outcome of public database datamined in our study [13, 20, 34], but a paradoxical viewpoint was also reported [14]. With respect to other MT isoforms in GC, pan's group also did parts of the work, but no significant difference was found between tumor and nontumor tissue and even no MT1B expression was detected in gastric cells and tissues [34]. Taken together, relatively limited studies focused on MT family, especially individual MT isoform in GC and more researches are needed to make specific conclusions for each MT isoform in GC.

MTs overexpression was frequently reported to be associated with poor prognosis in a wide range of human cancers, such as hepatocellular carcinoma, breast cancer, glioblastoma, oral cancer, and melanoma [23, 35–38]. Although the prognostic role of MTs in gastric cancer was also evaluated in several studies, it is still hard to conclude what exact value they possessed. For instance, several studies argued that no association was discovered among MT expression and tumor stage, differentiation, and survival prognosis in gastric cancer [14, 18, 32], while another research demonstrated that MT overexpression was associated with a poor survival rate [17]. In the current study, data showed that some specific MT isoforms like MT1F, MT1G, MT1H, and MT1X were associated with OS. Notably, the result reported by one group showed that loss of MT2A was associated with poor prognosis and advanced TNM stage,  , which was not in accordance with the findings of our study [13, 19, 20].

The broad heterogeneity of MT expression and its prognostic role in gastric cancer can be simplistically attributed to shortage of case numbers and different ethnicity of patients recruited or distinct transcripts of MT gene adopted in their studies. However, more important reason may be the facts that MT exists as a mixture of variable forms. Because of the high structure similarity of MTs, present proteomic methods lack the ability to distinguish all subisoforms. For example, the antibodies used in many studies could not specify MT1 and MT2 isoform due to their physical-chemical homology [39]. This speculation seems to be verified by the phenomenon mentioned above that the change in MT1/2 protein expression was different from single MT isoforms and different MT isoform plays distinct function in cell activities [38, 39]. Although the antibodies specific to MT1A, MT1G, and MT3 were available in market [9, 40], distinguishing of MTs by using antibodies is even more tricky.

DNA methylation is an important epigenetic modification in cancer formation by silencing tumor suppressor genes. A wide range of studies investigated MTs promoter methylation in some cancer types, but limiting studies about MTs methylation in gastric cancer were published up to now [21, 41–43]. In line with MT3 hypermethylation in gastric cancer showed by Deng et al., our study also showed that MT3 was highly hypermethylated compared with normal gastric tissue [33]. In the present study, we demonstrated that half of MT isoforms in GC were highly methylated in the promotor region except MT1E and that there was an inverse correlation between DNA methylation and mRNA expression of most isoforms of MT other than MT1A and MT4 isoforms. Therefore, it is not difficult to conclude that methylation in MTs gene promoter region partially contributed to their reduced expression in GC. In addition, our study revealed that MT genes are very rarely mutated and no statistically significant difference was observed for OS and disease-free survival (DFS) between cases with and without alteration of MTs in gastric cancer. The Epstein-Barr Virus (EBV), the second pathogen associated with GC, was found in approximately 20% of the samples in the present study, which was similar to previous studies [44–46]. Meanwhile, the outcomes in our study that GC located most frequently at the antrum are in line with previous studies [47–49]. Moreover, we established networks related to MT family to explore other genes involved in regulatory relationship between them.

In summary, all these findings indicated that MTs were nearly downregulated in GC tissue and their prognostic values in GC were dependent on single isoform of MT, which need to be determined further in de facto cohort studies. As such, our study offered comprehensive evidences to evaluate the possible regulating function of MTs in GC which may help for further discovering MTs as potential diagnostic or prognostic biomarkers and therapeutic target for GC in the future.

## 5. Study Limitations

However, the present study was not without limitations. First, all the data analyzed in our study was obtained from different online databases, which might cause background heterogeneity. Additionally, the study did not conduct experiments to validate the results obtained from silicobioinformatic analysis based on online databases. Therefore, more elaborate studies with focus on various MT isoforms expression and prognostic value in GC need to be performed in the future.

## Figures and Tables

**Table 1:** The transcription levels of MT family isoforms between different types of GC and normal tissues (ONCOMINE).

MT family members	Types of GC vs. normal	Fold change	*t*-Test	*P* value	Reporter
MT1A	Gastric Intestinal Type Adenocarcinoma vs. Normal	-2.939	-5.52	1.48E-06	ILMN-1691156
	Diffuse Gastric Adenocarcinoma vs. Normal	-3.151	-6.36	1.04E-07	ILMN-1691156
	Gastric Mixed Adenocarcinoma vs. Normal	-3.053	-3.47	2.00E-03	ILMN-1691156
	Gastric Adenocarcinoma vs. Normal	-2.255	-0.99	**0.196**	ILMN-1691156

MT1B	Gastric Intestinal Type Adenocarcinoma vs. Normal	-2.496	-7.43	3.42E-09	IMAGE:232772
	Diffuse Gastric Adenocarcinoma vs. Normal	-2.037	-4.43	5.52E-05	IMAGE:232772
	Gastric Mixed Adenocarcinoma vs. Normal	-2.321	-4.43	2.26E-04	IMAGE:232772
	Gastric Cancer vs. Normal	**-1.327** ^▲^	-1.65	**0.051**	3662190
	Gastric Mixed Adenocarcinoma vs. Normal	**-1.111** ^▲^	-1.31	**0.102**	ILMN-1733758
	Gastric Intestinal Type Adenocarcinoma vs. Normal	**-1.065** ^▲^	-0.73	**0.236**	ILMN-1733758
	Diffuse Gastric Adenocarcinoma vs. Normal	**1.025** ^**∗**^	0.223	**0.588**	ILMN-1733758
	Gastric Adenocarcinoma vs. Normal	**1.326** ^**∗**^	0.793	**0.76**	ILMN-1733758

MT1E	Gastric Mixed Adenocarcinoma vs. Normal	-3.006	-7.8	1.16E-08	ILMN-1718968
	Gastric Intestinal Type Adenocarcinoma vs. Normal	-3.014	-7.81	1.99E-09	ILMN-1718968
	Diffuse Gastric Adenocarcinoma vs. Normal	-3.042	-8.23	6.09E-10	ILMN-1718968
	Gastric Adenocarcinoma vs. Normal	-2.524	-4.05	0.005	ILMN-1718968
	Gastric Cancer vs. Normal	-3.942	-3.17	9.14E-04	3662139
	Gastric Mixed Adenocarcinoma vs. Normal	-5.468	-5.12	0.005	212859-x-at
	Gastric Cancer vs. Normal	-2.435	-2.79	0.006	212859-x-at
	Diffuse Gastric Adenocarcinoma vs. Normal	**-1.97** ^▲^	-2.66	0.018	212859-x-at
	Gastric Intestinal Type Adenocarcinoma vs. Normal	**-1.74** ^▲^	-3.17	0.001	212859-x-at

MT1F	Gastric Intestinal Type Adenocarcinoma vs. Normal	-5.011	-10.9	7.66E-14	IMAGE:78353
	Diffuse Gastric Adenocarcinoma vs. Normal	-3.821	-6.8	6.50E-08	IMAGE:78353
	Gastric Mixed Adenocarcinoma vs. Normal	-4.273	-4.69	3.35E-04	IMAGE:245990
	Diffuse Gastric Adenocarcinoma vs. Normal	-4.486	-8.37	2.13E-10	ILMN-1718766
	Gastric Intestinal Type Adenocarcinoma vs. Normal	-3.911	-6.22	1.69E-07	ILMN-1718766
	Gastric Mixed Adenocarcinoma vs. Normal	-4.355	-3.73	0.001	ILMN-1718766
	Gastric Mixed Adenocarcinoma vs. Normal	-4.283	-10.5	4.58E-06	213629-x-at
	Diffuse Gastric Adenocarcinoma vs. Normal	-2.712	-4.1	0.003	213629-x-at
	Gastric Intestinal Type Adenocarcinoma vs. Normal	-2.095	-4.22	5.69E-05	217165-x-at
	Gastric Cancer vs. Normal	-3.148	-3.12	0.003	213629-x-at
	Gastric Adenocarcinoma vs. Normal	-2.514	-0.81	**0.237**	ILMN-1718766

MT1G	Gastric Cancer vs. Normal	-3.231	-6.53	4.28E-10	3692999
	Diffuse Gastric Adenocarcinoma vs. Normal	-4.274	-6.82	4.25E-08	IMAGE:202535
	Gastric Intestinal Type Adenocarcinoma vs. Normal	-5.68	-10	1.04E-12	IMAGE:202535
	Gastric Mixed Adenocarcinoma vs. Normal	-5.508	-5.68	7.17E-05	IMAGE:202535
	Diffuse Gastric Adenocarcinoma vs. Normal	-4.092	-6.15	1.43E-04	204745-x-at
	Gastric Intestinal Type Adenocarcinoma vs. Normal	-2.636	-5.41	1.02E-06	204745-x-at
	Gastric Mixed Adenocarcinoma vs. Normal	-6.734	-8.01	3.57E-04	204745-x-at
	Diffuse Gastric Adenocarcinoma vs. Normal	-8.187	-8.02	1.19E-10	ILMN-1715401
	Gastric Intestinal Type Adenocarcinoma vs. Normal	-6.637	-6.24	2.22E-07	ILMN-1715401
	Gastric Mixed Adenocarcinoma vs. Normal	-4.742	-3.31	0.003	ILMN-1715401
	Gastric Cancer vs. Normal	-4.655	-3.41	0.001	210472-at
	Gastric Adenocarcinoma vs. Normal	-3.824	-1.15	**0.165**	ILMN-1715401

MT1H	Gastric Intestinal Type Adenocarcinoma vs. Normal	-4.137	-9.59	1.06E-12	IMAGE:214162
	Diffuse Gastric Adenocarcinoma vs. Normal	-3.314	-5.99	1.13E-06	IMAGE:214162
	Gastric Mixed Adenocarcinoma vs. Normal	-4.151	-4.5	8.45E-04	IMAGE:214162
	Diffuse Gastric Adenocarcinoma vs. Normal	-3.076	-5.1	4.59E-04	206461-x-at
	Gastric Mixed Adenocarcinoma vs. Normal	-4.261	-7.53	1.72E-04	206461-x-at
	Diffuse Gastric Adenocarcinoma vs. Normal	-6.768	-6.77	5.16E-08	ILMN-2124802
	Gastric Intestinal Type Adenocarcinoma vs. Normal	-5.882	-5.4	2.04E-06	ILMN-2124802
	Gastric Mixed Adenocarcinoma vs. Normal	-4.122	-2.75	0.008	ILMN-2124802
	Gastric Intestinal Type Adenocarcinoma vs. Normal	**-1.84** ^▲^	-3.76	2.21E-04	206461-x-at
	Gastric Adenocarcinoma vs. Normal	**-2.701**	-0.85	**0.228**	ILMN-2124802
	Gastric Cancer vs. Normal	**-1.32** ^▲^	-2.25	0.013	2462589

MT1M	Gastric Cancer vs. Normal	-4.451	-7.56	1.68E-12	3662150
	Gastric Intestinal Type Adenocarcinoma vs. Normal	-5.442	-11.2	8.37E-17	IMAGE:126458
	Diffuse Gastric Adenocarcinoma vs. Normal	-3.75	-6.3	9.03E-07	IMAGE:126458
	Gastric Mixed Adenocarcinoma vs. Normal	-6.244	-6.14	1.15E-04	IMAGE:126458
	Diffuse Gastric Adenocarcinoma vs. Normal	-8.169	-9	7.18E-10	ILMN-1657435
	Gastric Intestinal Type Adenocarcinoma vs. Normal	-5.42	-6.12	2.48E-07	ILMN-1657435
	Gastric Mixed Adenocarcinoma vs. Normal	-3.749	-3.03	0.004	ILMN-1657435
	Gastric Intestinal Type Adenocarcinoma vs. Normal	-5.088	-6.1	2.23E-07	217546-at
	Diffuse Gastric Adenocarcinoma vs. Normal	-4.402	-3.57	0.006	217546-at
	Gastric Cancer vs. Normal	-10.35	-3.32	0.003	217546-at
	Gastric Adenocarcinoma vs. Normal	**-1.999** ^▲^	-0.87	**0.221**	ILMN-1657435

MT1X	Gastric Intestinal Type Adenocarcinoma vs. Normal	-3.75	-9.04	8.45E-12	IMAGE:297392
	Diffuse Gastric Adenocarcinoma vs. Normal	-2.85	-5.07	1.17E-05	IMAGE:297392
	Gastric Mixed Adenocarcinoma vs. Normal	-3.223	-4.26	6.15E-04	IMAGE:297392
	Gastric Intestinal Type Adenocarcinoma vs. Normal	-4.098	-6.29	1.63E-07	ILMN-1775170
	Diffuse Gastric Adenocarcinoma vs. Normal	-3.782	-6.42	1.60E-07	ILMN-1775170
	Gastric Mixed Adenocarcinoma vs. Normal	-3.21	-3.71	8.60E-04	ILMN-1775170
	Diffuse Gastric Adenocarcinoma vs. Normal	-2.371	-4.44	0.001	208581-x-at
	Gastric Mixed Adenocarcinoma vs. Normal	-3.11	-4.86	0.004	208581-x-at
	Gastric Cancer vs. Normal	-3.261	-4.92	1.09E-06	3662247
	Gastric Adenocarcinoma vs. Normal	-2.329	-1.1	**0.172**	ILMN-1775170
	Gastric Intestinal Type Adenocarcinoma vs. Normal	**-1.55** ^▲^	-2.95	0.002	208581-x-at
	Gastric Cancer vs. Normal	**-1.99** ^▲^	-2.18	**0.021**	208581-x-at

MT2A	Diffuse Gastric Adenocarcinoma vs. Normal	-2.117	-4.32	0.001	212185-x-at
	Diffuse Gastric Adenocarcinoma vs. Normal	-2.308	-4.56	2.50E-05	ILMN-1686664
	Gastric Intestinal Type Adenocarcinoma vs. Normal	-2.153	-3.85	2.25E-04	ILMN-1686664
	Gastric Mixed Adenocarcinoma vs. Normal	-2.68	-3.67	9.96E-04	ILMN-1686664
	Gastric Intestinal Type Adenocarcinoma vs. Normal	**-1.42** ^▲^	-2.7	0.005	208581-x-at
	Gastric Adenocarcinoma vs. Normal	**-1.89** ^▲^	-0.86	**0.224**	ILMN-1686664
	Gastric Cancer vs. Normal	**-1.87** ^▲^	-2.33	0.015	212185-x-at

MT3	Gastric Intestinal Type Adenocarcinoma vs. Normal	-2.85	-8.47	1.47E-10	IMAGE:2019011
	Diffuse Gastric Adenocarcinoma vs. Normal	-2.312	-5.22	5.48E-06	IMAGE:2019011
	Gastric Mixed Adenocarcinoma vs. Normal	-2.688	-4.68	2.23E-04	IMAGE:2019011
	Diffuse Gastric Adenocarcinoma vs. Normal	**-1.21** ^▲^	-3.71	3.47E-04	ILMN-1675947
	Gastric Intestinal Type Adenocarcinoma vs. Normal	**-1.14** ^▲^	-1.89	0.034	ILMN-1675947
	Gastric Mixed Adenocarcinoma vs. Normal	**-1.11** ^▲^	-0.77	**0.229**	ILMN-1675947
	Gastric Adenocarcinoma vs. Normal	**1.107** ^**∗**^	0.315	**0.614**	ILMN-1675947
	Gastric Cancer vs. Normal	**-1.24** ^▲^	-2.81	0.003	3662093
	Diffuse Gastric Adenocarcinoma vs. Normal	**-1.79** ^▲^	-1.63	**0.079**	205970-at
	Gastric Mixed Adenocarcinoma vs. Normal	**-1.97** ^▲^	-2.04	0.061	205970-at
	Gastric Intestinal Type Adenocarcinoma vs. Normal	**-1.21** ^▲^	-1.33	0.096	205970-at
	Gastric Cancer vs. Normal	**-1.19** ^▲^	-0.57	**0.288**	205970-at

MT4	Gastric Cancer vs. Normal	-2.723	-5.62	5.26E-08	3662086
	Gastric Mixed Adenocarcinoma vs. Normal	**1.02** ^**∗**^	0.673	**0.744**	ILMN-1745345
	Diffuse Gastric Adenocarcinoma vs. Normal	**1.018** ^**∗**^	1.026	**0.844**	ILMN-1745345
	Gastric Intestinal Type Adenocarcinoma vs. Normal	**1.032** ^**∗**^	1.191	**0.879**	ILMN-1745345
	Gastric Adenocarcinoma vs. Normal	**1.461** ^**∗**^	1.104	**0.825**	ILMN-1745345

*Notes: P* value was analyzed using the t-test. The bold font indicates that the difference was not statistically significant between the GC and normal tissue group. The bold font with symbol “^▲^” indicates the fold change was no more than 2 folds. The bold font with symbol “^*∗*^” indicates the transcription level of MTs in gastric cancer was slightly higher than normal tissue.

**Table 2:** The prognostic values of MT isoforms in different pathological subtypes of GC patients (Kaplan-Meier plotter).

MT family	Lauren classification	OS				PPS			
		cases	HR	95%CI	*P* value	cases	HR	95%CI	*P* value
MT1E	intestinal	320	0.67	0.49-0.92	**0.013**	192	1.39	0.92-2.1	0.12
	diffuse	241	0.55	0.38-0.79	**0.0011**	176	0.53	0.34-0.83	**0.0045**
	mixed	32	5.92	0.77-45.35	0.053	16	–	–	–

MT1F	intestinal	320	0.63	0.46-0.88	**0.0056**	192	0.7	0.45-1.09	0.11
	diffuse	241	0.62	0.44-0.88	**0.0063**	176	0.48	0.3-0.77	**0.0019**
	mixed	32	2.02	0.68-5.99	0.2	16	–	–	–

MT1G	intestinal	320	1.99	1.42-2.8	**0.00005**	192	2.6	1.7-3.97	**4.3E-05**
	diffuse	241	1.83	1.18-2.82	**0.006**	176	1.53	1.03-2.28	**0.034**
	mixed	32	3.32	1.3-9.82	**0.021**	16	–	–	–

MT1H	intestinal	320	0.7	0.5-1	**0.046**	192	0.63	0.4-1.0	**0.047**
	diffuse	241	0.55	0.39-0.77	**0.00053**	176	0.54	0.36-0.79	**0.0015**
	mixed	32	0.33	0.07-1.47	0.13	16	–	–	–

MT1M	intestinal	320	0.59	0.42-0.84	**0.0027**	192	1.48	0.98-2.25	0.061
	diffuse	241	0.59	0.39-0.89	**0.011**	176	0.54	0.34-0.84	**0.0051**
	mixed	32	1.57	0.57-4.36	0.38	16	–	–	–

MT1X	intestinal	320	0.58	0.42-0.82	**0.0015**	192	0.65	0.42-0.99	**0.041**
	diffuse	241	0.57	0.4-0.79	**0.00087**	176	0.56	0.38-0.83	**0.0029**
	mixed	32	0.49	0.14-1.75	0.26	16	–	–	–

MT2A	intestinal	320	1.27	0.93-1.75	0.13	192	1.49	0.99-2.25	0.055
	diffuse	241	0.59	0.41-0.86	**0.0047**	176	0.65	0.44-0.97	**0.035**
	mixed	32	0.52	0.18-1.51	0.22	16	–	–	–

MT3	intestinal	320	2.34	1.69-3.26	**1.8E-06**	192	1.79	1.18-2.7	**0.0051**
	diffuse	241	1.24	0.83-1.86	0.29	176	1.23	0.78-1.94	0.37
	mixed	32	1.43	0.49-4.2	0.51	16	–	–	–

MT4	intestinal	320	2.58	1.87-3.56	**1.8E-08**	192	2.48	1.4-4.4	**0.0013**
	diffuse	241	0.72	0.52-1.02	0.06	176	1.5	0.96-2.33	0.07
	mixed	32	2.8	1.01-7.77	**0.038**	16	–	–	–

*Notes: P* value was analyzed using the survival analysis test. The fold indicates that the difference was statically significant. The *P* value was set up at 0.05. *Abbreviations:* GC: gastric cancer; OS: overall survival; PPS: postprogression survival; HR: hazard ratio.

**Table 3:** The prognostic values of MT isoforms in GC patients with different clinical stage (Kaplan-Meier plotter).

MT family	clinical stage	OS				PPS			
		cases	HR	95%CI	*P* value	cases	HR	95%CI	*P* value
MT1E	I	67	0.43	0.16-1.16	0.085	31	0.1	0.02-0.71	**0.007**
	II	140	0.63	0.32-1.23	0.17	105	0.6	0.26-1.35	0.21
	III	305	0.72	0.54-0.96	**0.026**	142	0.7	0.45-1.1	0.12
	IV	148	0.72	0.46-1.14	1.14	104	1.3	0.84-2.07	0.22

MT1F	I	67	0.27	0.1-0.72	**0.005**	31	0.1	0.02-0.69	**0.006**
	II	140	0.54	0.27-1.08	0.075	105	0.6	0.27-1.41	0.25
	III	305	0.66	0.49-0.88	**0.004**	142	0.7	0.44-1.03	0.068
	IV	148	0.7	0.44-1.11	0.12	104	1.3	0.77-2.25	0.31

MT1G	I	67	2.87	0.92-8.96	0.058	31	6.6	6.25-34.15	**0.011**
	II	140	1.69	0.88-3.23	0.11	105	2	0.79-5.24	0.13
	III	305	1.68	1.26-2.23	**4E-04**	142	2	1.29-3.02	**0.001**
	IV	148	1.78	1.16-2.73	**0.007**	104	2.4	1.46-3.97	**4E-04**

MT1H	I	67	2	0.71-5.57	0.18	31	0.2	0.02-1.79	0.12
	II	140	0.54	0.28-1.04	0.061	105	0.5	0.25-1.07	0.07
	III	305	0.75	0.57-1.01	0.054	142	0.7	0.45-1.04	0.076
	IV	148	0.62	0.41-0.95	**0.027**	104	1.3	0.74-2.1	0.41

MT1M	I	67	2.34	0.67-8.23	0.17	31	2.8	0.55-14.59	0.2
	II	140	0.82	0.44-1.52	0.52	105	0.7	0.37-1.42	0.35
	III	305	0.73	0.52-1.02	0.067	142	0.7	0.45-1.11	0.13
	IV	148	0.73	0.46-1.15	0.17	104	1.5	0.92-2.32	0.11

MT1X	I	67	0.29	0.1-0.8	**0.011**	31	0	—	**0.002**
	II	140	0.49	0.22-1.11	0.081	105	0.6	0.29-1.33	0.22
	III	305	0.61	0.46-0.81	**7E-04**	142	0.6	0.39-0.92	**0.017**
	IV	148	0.66	0.45-0.99	**0.041**	104	1.4	0.79-2.35	0.27

MT2A	I	67	0.35	0.13-0.93	**0.028**	31	0.1	0.02-0.67	**0.005**
	II	140	0.57	0.28-1.16	0.12	105	0.6	0.27-1.43	0.26
	III	305	0.79	0.59-1.05	0.11	142	0.7	0.47-1.16	0.19
	IV	148	0.78	0.51-1.18	0.24	104	1.5	0.93-2.27	0.095

MT3	I	67	1.79	0.61-5.21	0.28	31	3.5	0.76-16.1	0.089
	II	140	2.07	1.14-3.74	**0.014**	105	2	0.99-3.82	**0.049**
	III	305	2.05	1.48-2.83	**1E-05**	142	2.1	1.35-3.22	**7E-04**
	IV	148	1.38	0.89-2.14	0.14	104	0.6	0.35-0.93	**0.023**

MT4	I	67	1.87	0.69-5.04	0.21	31	2.7	0.32-23.29	0.34
	II	140	1.3	0.62-2.71	0.49	105	1.7	0.81-3.7	0.15
	III	305	1.88	1.41-2.52	**2E-05**	142	1.9	1.21-2.95	**0.004**
	IV	148	1.29	0.88-1.91	0.19	104	1.3	0.81-2.04	0.29

*Notes: P* value was analyzed using the survival analysis test. The fold indicates that the difference was statically significant. The *P* value was set up at 0.05. *Abbreviations:* GC: gastric cancer; OS: overall survival; PPS: postprogression survival; HR: hazard ratio.

**Table 4:** The correlation between DNA methylation and mRNA expression in the MT gene members of GC patients (MethHC).

Variable	Methylation										
	MT1A	MT1B	MT1E	MT1F	MT1G	MT1H	MT1M	MT1X	MT2A	MT3	MT4
mRNA expression	r=0.007	r=-0.123	r=-0.279	r=-0.116	r=-0.158	r=-0.230	r=-0.187	r=0.010	r=-0.160	r=-0.248	r=-0.02
	*p*=4.44	*p<*0.001^*∗*^	*p<*0.001^*∗*^	*p<*0.001^*∗*^	*p<*0.001^*∗*^	*p<*0.001^*∗*^	*p<*0.001^*∗*^	*p<*0.001^*∗*^	*p<*0.001^*∗*^	*p<*0.001^*∗*^	p=0.59

*Notes:* “^*∗*^” indicates statistical significance with *P*<0.001. *Abbreviation:* GC: gastric cancer.

## Data Availability

All data like figures and tables used to support the findings of this study are included within the article. All data like supplementary figures and tables used to support the findings of this study are included within the supplementary information materials. All unavailable data used to support the findings of this study can be seen on related database online. We did not release them not because we did not give the original data but we gave it in another form which could not reflect it directly.

## Supplementary Materials

Supplementary Materials Supplementary Figure 1: the correlation between the expression of MTs and tumor stages in GC patients (GEPIA). Supplementary Figure 2: the prognostic values with no significance of mRNA level of MTs in all GC patients (SurvExpress, Kaplan-Meier plotter). Supplementary Tables 1–3: the prognostic values of MT isoforms in GC patients with different differentiation, HER2 status, and treatment (Kaplan-Meier plotter).
