# Supplementary material for: Evaluation of MT Family Isoforms as Potential Biomarker for Predicting Progression and Prognosis in Gastric Cancer
Source: Biomed Res Int. 2019 Jul 17;2019:2957821. doi: 10.1155/2019/2957821 (PMC6662468; doi:10.1155/2019/2957821)
Supplement: Supplementary Materials — Supplementary Figure 1: the correlation between the expression of MTs and tumor stages in GC patients (GEPIA). Supplementary Figure 2: the prognostic values with no significance of mRNA level of MTs in all GC patients (SurvExpress, Kaplan-Meier plotter). Supplementary Tables 1–3: the prognostic values of MT isoforms in GC patients with different differentiation, HER2 status, and treatment (Kaplan-Meier plotter). [file 2957821.f1.docx]

**Supplementary Figure 1** The correlation between the expression of MTs and tumor stages in GC patients (GEPIA)


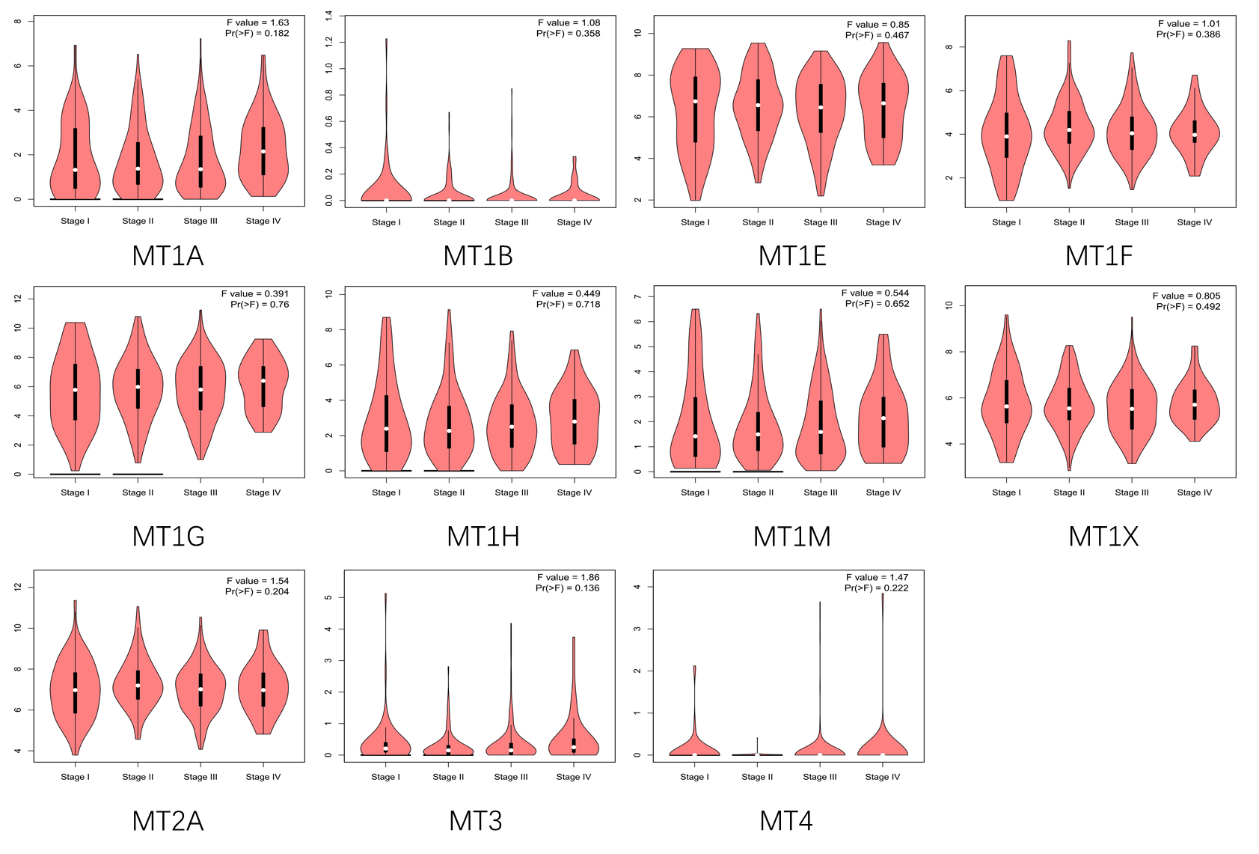


**abbreviation:** GC: gastric cancer.

**Supplementary Figure 2.** The prognostic values of mRNA level of MTs in GC patients (SurvExpress, Kaplan-Meier plotter)


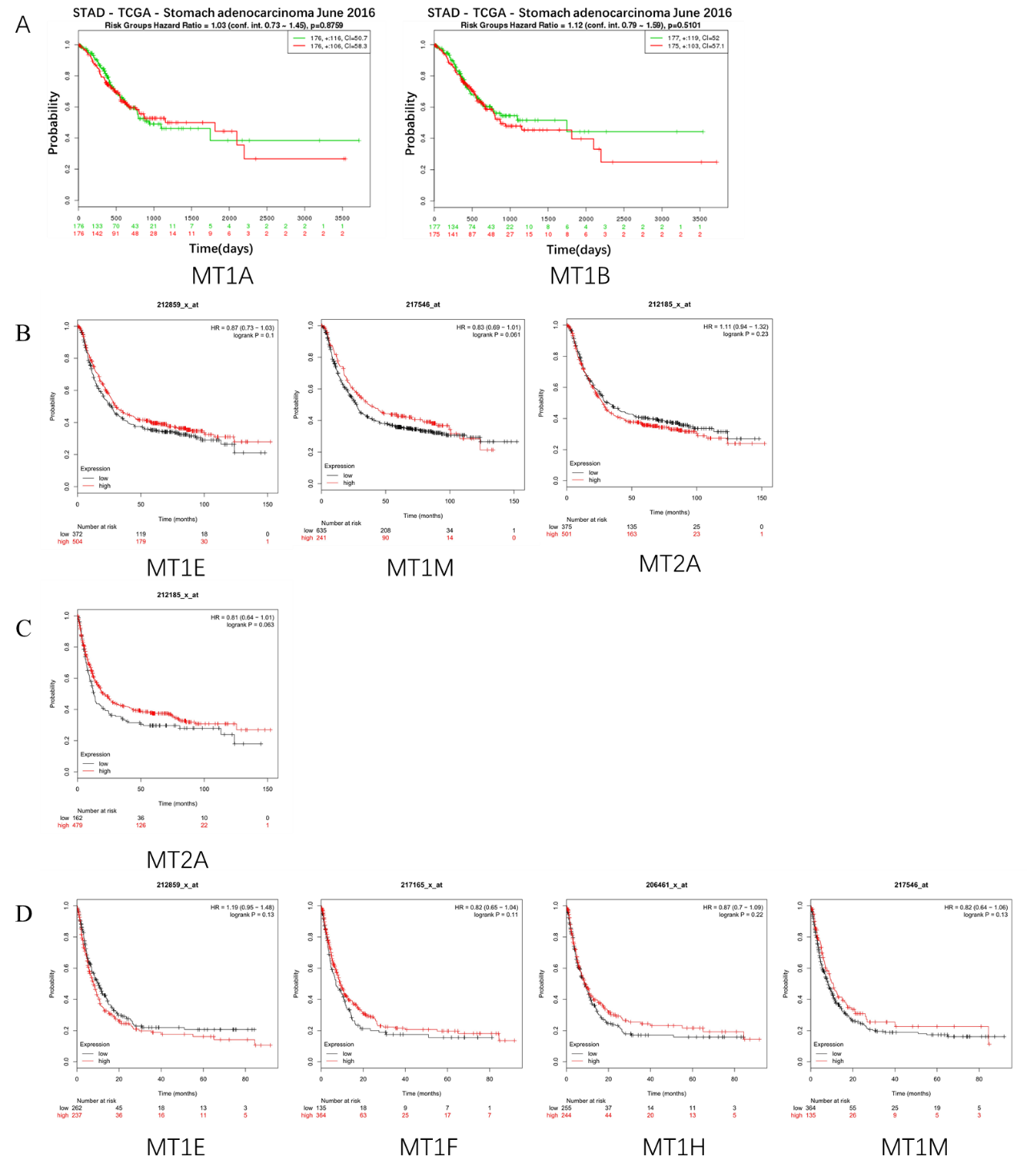


**Notes:** The association between the members of MT family and OS, FP and PPS in GC patients respectively with no statistical significance from SurvExpress (**A**) and Kaplan-Meier plotter (**B-D**). **A:** OS curves of MT1A and MT1B; **B:** OS curves of MT1E (Affymetrix ID:212859_-_x at); MT1M (Affymetrix ID:217546_-_x at); MT2A (Affymetrix ID:212185_-_x at). **C:** FP curves of MT2A. **D:** PPS curves of MT1E; MT1F (Affymetrix ID:217165_-_x at); MT1H (Affymetrix ID:206461_-_x at); MT1M (Affymetrix ID:217546_-_x at). **Abbreviations:** OS: overall survival; FP: first progression; PPS: post progression survival; GC: gastric cancer; HR: hazard ratio.

**Supplementary Table 1.** The prognostic values of MT isoforms in GC patients with different differentiation (Kaplan-Meier plotter)

| MT family | differentiation | OS | | | | PPS | | | |
| --- | --- | --- | --- | --- | --- | --- | --- | --- | --- |
|  |  | cases | HR | 95%CI | *p*-value | cases | HR | 95%CI | *p*-value |
| MT1E | poorly differentiated | 165 | 0.7 | 0.47-1.05 | 0.081 | 49 | 0.46 | 0.21-1.03 | 0.054 |
|  | moderately differentiated | 67 | 0.41 | 0.2-0.81 | **0.0085** | 24 | 1.56 | 0.62-3.95 | 0.34 |
|  | well differentiated | 32 | 1.99 | 0.84-4.73 | 0.11 | 0 | – | – | – |
| MT1F | poorly differentiated | 165 | 0.61 | 0.39-0.96 | **0.031** | 49 | 0.55 | 0.26-1.15 | 0.11 |
|  | moderately differentiated | 67 | 0.5 | 0.26-0.97 | **0.036** | 24 | 1.79 | 0.66-4.85 | 0.24 |
|  | well differentiated | 32 | 0.5 | 0.21-1.19 | 0.11 | 0 | – | – | – |
| MT1G | poorly differentiated | 165 | 0.8 | 0.54-1.19 | 0.28 | 49 | 2.01 | 0.84-4.83 | 0.11 |
|  | moderately differentiated | 67 | 0.4 | 0.17-0.96 | **0.034** | 24 | 0.31 | 0.11-0.85 | **0.016** |
|  | well differentiated | 32 | 4.09 | 0.95-17.66 | **0.041** | 0 | – | – | – |
| MT1H | poorly differentiated | 165 | 0.6 | 0.39-0.9 | **0.013** | 49 | 0.44 | 0.2-0.99 | **0.041** |
|  | moderately differentiated | 67 | 0.59 | 0.3-1.15 | 0.12 | 24 | 1.64 | 0.62-4.35 | 0.32 |
|  | well differentiated | 32 | 0.64 | 0.27-1.52 | 0.31 | 0 | – | – | – |
| MT1M | poorly differentiated | 165 | 0.6 | 0.38-0.95 | **0.028** | 49 | 0.38 | 0.19-0.76 | **0.0049** |
|  | moderately differentiated | 67 | 0.58 | 0.28-1.18 | 0.13 | 24 | 0.65 | 0.23-1.85 | 0.41 |
|  | well differentiated | 32 | 1.61 | 0.68-3.83 | 0.28 | 0 | – | – | – |
| MT1X | poorly differentiated | 165 | 0.77 | 0.51-1.16 | 0.21 | 49 | 0.51 | 0.26-1.03 | 0.056 |
|  | moderately differentiated | 67 | 0.39 | 0.16-0.95 | **0.031** | 24 | 2.02 | 0.71-5.77 | 0.18 |
|  | well differentiated | 32 | 0.6 | 0.25-1.45 | 0.25 | 0 | – | – | – |
| MT2A | poorly differentiated | 165 | 0.66 | 0.44-0.98 | **0.039** | 49 | 0.44 | 0.21-0.95 | **0.033** |
|  | moderately differentiated | 67 | 0.37 | 0.16-0.86 | **0.015** | 24 | 0.57 | 0.21-1.56 | 0.27 |
|  | well differentiated | 32 | 0.69 | 0.25-1.89 | 0.47 | 0 | – | – | – |
| MT3 | poorly differentiated | 165 | 1.21 | 0.77-1.92 | 0.41 | 49 | 0.67 | 0.35-1.27 | 0.21 |
|  | moderately differentiated | 67 | 0.56 | 0.25-1.28 | 0.17 | 24 | 0.53 | 0.17-1.6 | 0.25 |
|  | well differentiated | 32 | 0.58 | 0.24-1.39 | 0.22 | 0 | – | – | – |
| MT4 | poorly differentiated | 165 | 1.34 | 0.9-1.99 | 0.15 | 49 | 1.74 | 0.91-3.33 | 0.09 |
|  | moderately differentiated | 67 | 0.62 | 0.29-1.31 | 0.21 | 24 | 1.56 | 0.59-4.14 | 0.36 |
|  | well differentiated | 32 | 2.65 | 0.78-9.06 | 0.11 | 0 | – | – | – |

**Notes:** *P*-value was analyzed using the survival analysis test. The fold indicates that the difference was statically significant. The *P*-value was set up at 0.05. **Abbreviations:** GC: gastric cancer; OS: overall survival; PPS: post progression survival; HR: hazard ratio.

**Supplementary Table 2.** The prognostic values of MT isoforms in GC patients with different HER2 status (Kaplan-Meier plotter)

| MT family | HER2 status | OS | | | | PPS | | | |
| --- | --- | --- | --- | --- | --- | --- | --- | --- | --- |
|  |  | cases | HR | 95%CI | *p*-value | cases | HR | 95%CI | *p*-value |
| MT1E | HER2 negative | 532 | 0.81 | 0.64-1.04 | 0.095 | 334 | 0.86 | 0.64-1.17 | 0.34 |
|  | HER2 positive | 344 | 1.31 | 1-1.73 | **0.049** | 165 | 1.4 | 0.99-1.99 | 0.054 |
| MT1F | HER2 negative | 532 | 0.71 | 0.55-0.91 | **0.0071** | 334 | 0.75 | 0.56-1 | **0.048** |
|  | HER2 positive | 344 | 0.8 | 0.6-1.06 | 0.11 | 165 | 1.29 | 0.91-1.84 | 0.15 |
| MT1G | HER2 negative | 532 | 2.04 | 1.54-2.7 | **3E-06** | 334 | 2.18 | 1.62-2.92 | **1E-07** |
|  | HER2 positive | 344 | 1.29 | 0.95-1.74 | 0.1 | 165 | 2.11 | 1.43-3.09 | **0.0001** |
| MT1H | HER2 negative | 532 | 0.74 | 0.59-0.93 | **0.01** | 334 | 0.78 | 0.58-1.03 | 0.08 |
|  | HER2 positive | 344 | 0.87 | 0.66-1.17 | 0.36 | 165 | 0.76 | 0.51-1.14 | 0.19 |
| MT1M | HER2 negative | 532 | 0.8 | 0.62-1.02 | 0.071 | 334 | 1.23 | 0.91-1.68 | 0.18 |
|  | HER2 positive | 344 | 1.18 | 0.91-1.53 | 0.22 | 165 | 1.4 | 0.92-2.12 | 0.11 |
| MT1X | HER2 negative | 532 | 0.63 | 0.5-0.79 | **0.0006** | 334 | 0.66 | 0.5-0.88 | **0.004** |
|  | HER2 positive | 344 | 0.75 | 0.58-0.98 | **0.033** | 165 | 0.78 | 0.55-1.11 | 0.16 |
| MT2A | HER2 negative | 532 | 0.9 | 0.71-1.14 | 0.39 | 334 | 1.42 | 1.07-1.89 | **0.015** |
|  | HER2 positive | 344 | 1.34 | 0.99-1.83 | 0.06 | 165 | 1.4 | 0.98-1.98 | 0.061 |
| MT3 | HER2 negative | 532 | 1.64 | 1.31-2.05 | **0.0002** | 334 | 1.63 | 1.21-2.21 | **0.0013** |
|  | HER2 positive | 344 | 1.45 | 1.08-1.94 | **0.014** | 165 | 1.79 | 1.16-2.75 | **0.0075** |
| MT4 | HER2 negative | 532 | 1.68 | 1.33-2.11 | **9E-05** | 334 | 2.38 | 1.76-3.22 | **8E-08** |
|  | HER2 positive | 344 | 1.4 | 1.04-1.9 | **0.027** | 165 | 1.55 | 1.08-2.24 | **0.017** |

**Notes:** *P*-value was analyzed using the survival analysis test. The fold indicates that the difference was statically significant. The *P*-value was set up at 0.05. **Abbreviations:** GC: gastric cancer; OS: overall survival; PPS: post progression survival; HR: hazard ratio.

**Supplementary Table 3.** The prognostic values of MT isoforms in GC patients with different treatment (Kaplan-Meier plotter)

| MT family | Treatment | OS | | | | PPS | | | |
| --- | --- | --- | --- | --- | --- | --- | --- | --- | --- |
|  |  | cases | HR | 95%CI | *p*-value | cases | HR | 95%CI | *p*-value |
| MT1E | surgery alone | 380 | 0.68 | 0.51-0.91 | **0.009** | 277 | 0.8 | 0.58-1.1 | 0.16 |
|  | 5 FU based adjuvant | 153 | 1.38 | 0.96-1.99 | 0.08 | 136 | 1.15 | .082-1.63 | 0.42 |
|  | other adjuvants | 76 | 0.47 | 0.14-1.61 | 0.22 | 74 | 0.3 | 0.07-1.31 | 0.09 |
| MT1F | surgery alone | 380 | 0.65 | 0.48-0.88 | **0.005** | 277 | 0.72 | 0.52-1 | 0.052 |
|  | 5 FU based adjuvant | 153 | 1.31 | 0.92-1.86 | 0.13 | 136 | 1.18 | 0.82-1.68 | 0.37 |
|  | other adjuvants | 76 | 0.33 | 0.1-1.14 | 0.065 | 74 | 0.33 | 0.09-1.14 | 0.065 |
| MT1G | surgery alone | 380 | 1.6 | 1.17-2.18 | **0.003** | 277 | 1.84 | 1.35-2.52 | **1E-04** |
|  | 5 FU based adjuvant | 153 | 1.45 | 0.98-2.13 | 0.061 | 136 | 1.47 | 0.98-2.2 | 0.059 |
|  | other adjuvants | 76 | 1.44 | 0.6-3.48 | 0.41 | 74 | 1.59 | 0.66-3.65 | 0.3 |
| MT1H | surgery alone | 380 | 0.71 | 0.53-0.95 | **0.019** | 277 | 0.72 | 0.51-1.01 | 0.055 |
|  | 5 FU based adjuvant | 153 | 1.33 | 0.91-1.95 | 0.14 | 136 | 1.15 | 0.81-1.63 | 0.43 |
|  | other adjuvants | 76 | 0.52 | 0.21-1.26 | 0.14 | 74 | 0.49 | 0.2-1.19 | 0.11 |
| MT1M | surgery alone | 380 | 0.85 | 0.61-1.18 | 0.34 | 277 | 1.24 | 0.9-1.72 | 0.18 |
|  | 5 FU based adjuvant | 153 | 0.55 | 0.37-0.82 | **0.003** | 136 | 0.65 | 0.45-0.93 | **0.017** |
|  | other adjuvants | 76 | 0.24 | 0.05-1.01 | **0.034** | 74 | 0.14 | 0.01-1.08 | **0.029** |
| MT1X | surgery alone | 380 | 0.66 | 0.49-0.88 | **0.005** | 277 | 0.63 | 0.46-0.87 | **0.005** |
|  | 5 FU based adjuvant | 153 | 1.23 | 0.85-1.78 | 0.27 | 136 | 0.73 | 0.49-1.09 | 0.12 |
|  | other adjuvants | 76 | 0.42 | 0.12-1.42 | 0.15 | 74 | 0.48 | 0.2-1.17 | 0.099 |
| MT2A | surgery alone | 380 | 0.69 | 0.5-0.93 | **0.015** | 277 | 1.34 | 0.98-1.83 | 0.068 |
|  | 5 FU based adjuvant | 153 | 1.38 | 0.96-1.99 | 0.085 | 136 | 0.82 | 0.57-1.16 | 0.26 |
|  | other adjuvants | 76 | 0.53 | 0.21-1.33 | 0.17 | 74 | 0.5 | 0.16-1.5 | 0.21 |
| MT3 | surgery alone | 380 | 1.48 | 1.09-2 | **0.011** | 277 | 1.53 | 1.1-2.13 | **0.011** |
|  | 5 FU based adjuvant | 153 | 1.27 | 0.89-1.82 | 0.18 | 136 | 1.43 | 0.95-2.15 | 0.081 |
|  | other adjuvants | 76 | 1.8 | 0.65-4.95 | 0.25 | 74 | 0.45 | 0.15-1.39 | 0.16 |
| MT4 | surgery alone | 380 | 1.4 | 0.99-2 | 0.059 | 277 | 2.15 | 1.42-3.27 | **2E-04** |
|  | 5 FU based adjuvant | 153 | 1.71 | 1.16-2.52 | **0.006** | 136 | 1.61 | 1.06-2.44 | **0.023** |
|  | other adjuvants | 76 | 2.11 | 0.86-5.16 | 0.096 | 74 | 2.64 | 1.07-6.51 | **0.029** |

**Notes:** *P*-value was analyzed using the survival analysis test. The fold indicates that the difference was statically significant. The *P*-value was set up at 0.05. **Abbreviations:** GC: gastric cancer; OS: overall survival; PPS: post progression survival; HR: hazard ratio.
